# Exploration of Raw Pigmented-Fleshed Sweet Potatoes Volatile Organic Compounds and the Precursors

**DOI:** 10.3390/molecules29030606

**Published:** 2024-01-26

**Authors:** Yanqiang Yao, Rong Zhang, Ruixue Jia, Zhufang Yao, Yake Qiao, Zhangying Wang

**Affiliations:** 1 College of Agriculture and Biotechnology, Hebei Normal University of Science & Technology, Changli 066600, China; yaoyanqiang2024@163.com; 2 Guangdong Province Key Laboratory of Crop Genetic Improvement, Crops Research Institute, Guangdong Academy of Agricultural Sciences, Guangzhou 510640, China; zr9362@126.com (R.Z.); jiaruixue91@126.com (R.J.); yaozhufang@gdaas.cn (Z.Y.)

**Keywords:** sweet potato, volatile organic compounds, key aromatic compounds, aroma precursors

## Abstract

Sweet potato provides rich nutrients and bioactive substances for the human diet. In this study, the volatile organic compounds of five pigmented-fleshed sweet potato cultivars were determined, the characteristic aroma compounds were screened, and a correlation analysis was carried out with the aroma precursors. In total, 66 volatile organic compounds were identified. Terpenoids and aldehydes were the main volatile compounds, accounting for 59% and 17%, respectively. Fifteen compounds, including seven aldehydes, six terpenes, one furan, and phenol, were identified as key aromatic compounds for sweet potato using relative odor activity values (ROAVs) and contributed to flower, sweet, and fat flavors. The OR sample exhibited a significant presence of *trans*-β-Ionone, while the Y sample showed high levels of benzaldehyde. Starch, soluble sugars, 20 amino acids, and 25 fatty acids were detected as volatile compounds precursors. Among them, total starch (57.2%), phenylalanine (126.82 ± 0.02 g/g), and fatty acids (6.45 μg/mg) were all most abundant in Y, and LY contained the most soluble sugar (14.65%). The results of the correlation analysis revealed the significant correlations were identified between seven carotenoids and *trans*-β-Ionone, soluble sugar and nerol, two fatty acids and hexanal, phenylalanine and 10 fatty acids with benzaldehyde, respectively. In general, terpenoids and aldehydes were identified as the main key aromatic compounds in sweet potatoes, and carotenoids had more influence on the aroma of OR than other cultivars. Soluble sugars, amino acids, and fatty acids probably serve as important precursors for some key aroma compounds in sweet potatoes. These findings provide valuable insights for the formation of sweet potato aroma.

## 1. Introduction

Sweet potato (*Ipomoea batata* L.) is the sixth largest food crop in the world, providing rich nutrients and bioactive substances for the human diet, which is conducive to promoting human health [1,2]. Sweet potato flesh appears white, yellow, orange, and purple in different colors due to the accumulation of pigment content. The diverse components and compositions of carotenoids contribute varying hues to yellow and orange sweet potatoes [3,4]. These varieties are highly advantageous for human health due to their elevated carotenoid content, which serves as a significant source of vitamin A [5,6]. Moreover, these sweet potatoes have gained recognition for their pronounced sweetness and aroma.

In recent years, there has been a growing body of research focused on aroma, with volatile organic compounds (VOCs) playing a pivotal role [7]. Rice contains a wide range of VOCs, such as aldehydes, ketones, esters, and alcohols, which collectively contribute to the complex aroma profile. However, variations in the type and proportion of these VOCs result in distinct aromatic characteristics among different rice varieties [8,9]. The main aroma components of pear were identified as aldehydes, esters, and alcohols by Wang et al., with variations in aroma observed among different varieties [10]. In addition, benzeneacetaldehyde, 2-pentylfuran, and *(E)-2-nonenal* present in raisins give them a floral, fruity, and roasted flavor [11]. The release of VOCs in plants is attributed to secondary metabolism, which arises from the metabolic conversion of carbohydrates, amino acids, and fatty acids [12,13]. The production of 1-octene-3-1 and *2-heptanol* was found to be associated with the degradation of fatty acids in sour cream [14]. The variation in pork flavor is associated with differences in fatty acid composition, specifically the presence of C18:1n9c, C22:6n3, and C18:3n3 as key precursors contributing to the development of robust flavor profiles in pork [15]. The amino acids exhibited significant correlations with volatile components, and a positive correlation was observed between glycine and esters as well as total volatile compounds in kiwi wine [16]. Furthermore, it was discovered the accumulation and degradation of carotenoids in tea yielded a diverse range of carotenoid derivatives, which significantly contributed to the rich taste profile of tea [17]. Notably, α/β-ionone and dihydroactinidiolide are identified as key compounds responsible for imparting a fruity aroma to tea [18]. In our previous studies, it was determined the VOCs present in sweet potato primarily consisted of terpenoids and aldehydes [19]. However, the composition and the relationship between characteristic aroma and precursors of sweet potato have not been reported.

Therefore, this study determined the VOCs of five different colors of sweet potato cultivars, and further determined the content of possible aroma precursors (starch, soluble sugar, amino acids, fatty acids, and carotenoids). The purpose of this study was to identify the composition of VOCs in sweet potato and screen out the key aromatic compounds of sweet potato. Through correlation analysis between aroma precursors and key aromatic compound, we investigated the primary aroma precursors present in sweet potato. The results of this study will provide a new perspective for further understanding of sweet potato quality.

## 2. Results

### 2.1. Analysis of Volatile Organic Compounds for Five Sweet Potato Cultivars

**Figure 1:**
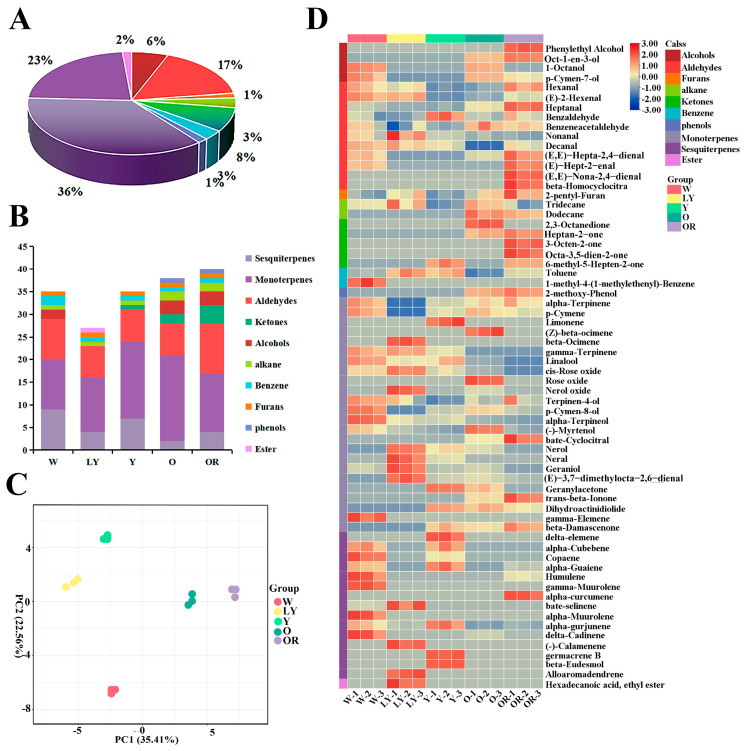
Analysis of the volatile organic compounds in five sweet potato cultivars. (**A**) Category statistics for volatile organic compounds. (**B**) Volatile organic compound composition statistics of five sweet potato cultivars. (**C**) The PCA score plots, each dot represents an independent experimental repeat. (**D**) Heatmap visualization of all the volatile organic compounds. The red blocks indicate the up-regulated metabolites, blue blocks indicate down-regulated metabolites, skin color blocks represent the average relative expressed intensity of all volatile organic compounds.

VOCs are important components of food flavor. In this study, the VOCs of five sweet potato cultivars were detected by GC-MS and a total of sixty-six VOCs were identified (Table S3). According to their structure, the compounds were divided into 10 classes, including alcohols, aldehydes, furans, alkanes, ketones, benzene, phenols, monoterpenes, sesquiterpenes, and esters. Obviously, terpenoids (monoterpenes and sesquiterpenes) and aldehydes were the main VOCs for sweet potato, which accounted for 59% (monoterpenes 36% and sesquiterpenes 23%) and 17% for the number of total VOCs (Figure 1A). In five cultivars, OR contained the greatest number of VOCs (40), followed by 38 in O, 35 in both W and Y, and at least 27 in LY (Figure 1B). Based on the accumulation levels of different VOCs, the principal component analysis (PCA) was performed. As indicated in Figure 1C, the total cumulative variance of 57.97% was contributed to by the two principal components (35.41% and 22.56%) from VOCs and the five sweet potato cultivars were well separated, which indicated there were significant differences among their VOCs.

To screen the difference of VOCs contents in the five sweet potato cultivars, a heatmap was constructed based on their relative content (Figure 1D) and one-way ANOVA analysis was performed (Table S3). Heatmaps show the presence of a large number of alcohols (Phenylethyl Alcohol, Oct-1-en-3-ol), aldehydes (*(E,E)-Hepta-2,4-dienal, (E)-Hept-2-enal, (E,E)-Nona-2,4-dienal*, and β-Homocyclocitra), ketones (Heptan-2-one, 3-Octen-2-one, and Octa-3,5-dien-2-one), and three monoterpenes (β-Cyclocitral, *trans*-β-Ionone, and β-Damascenone) in OR (Table S3). It was worth noting the relative contents of *trans*-β-Ionone and β-Damascenone increased gradually with the deepening of sweet potato flesh color, reaching their highest levels in OR. In O, alcohols, ketones, and a small quantity of monoterpenes were detected, with a notably high relative content observed for octan-2,3-dione (Table S3). The concentration of monoterpenes and sesquiterpenes in Y was relatively low, while the relative content of benzaldehyde was significantly higher compared to the other four cultivars (Table S3). LY exhibited a large number of monoterpenes, including nerol, neral, geaniol, and *(E)-3,7-dimethylocta-2,6-dienal*. W had a limited quantity of aldehydes, monoterpenes, and a substantial amount of sesquiterpenes (Table S3).

### 2.2. Analysis of Odor Activity Value of Five Sweet Potato Cultivars

**Figure 2:**
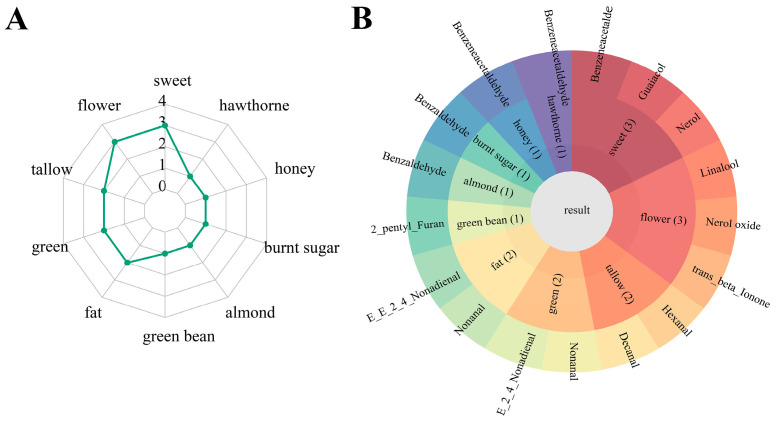
Composition of sweet potato characteristic aromas. (**A**) A radar map explains sweet potato flavor composition. (**B**) The pie chart explained the flavor composition contributed by characteristic aromas. The numbers in brackets represent the number of key aromatic compounds.

To comprehensively understand the contribution of VOCs to the aroma profile of sweet potato, we conducted an assessment of the relative odor activity value (ROAV) for VOCs in five distinct cultivars (Table 1). The results revealed among the identified VOCs, 15 exhibited an ROAV greater than 1. Specifically, this included seven aldehydes, one furan and phenol compound, as well as six terpenoids which contributed to the aroma profile of sweet potato. The radar map results indicate the presence of ten primary sweet potato aromas, with flower, sweet, tallow, green, and fat notes being the most prominent (Figure 2A). Figure 2B illustrates linalool, nerol oxide, and *trans*-β-ionone are responsible for giving sweet potatoes their flower aroma, with W, LY, and OR having the highest abundance, respectively (Table 1). Nerol, guaiacol, and benzeneacetaldehyde are the primary components that contribute to the sweet taste of sweet potatoes. The highest ROAV of nerol was found in Y, while benzeneacetaldehyde and guaiacol had the highest ROAV in O and OR, respectively (Table 1). The compounds hexanal and decanal exhibit the highest ROAV in LY, providing a tallow flavor to sweet potatoes. Nonanal and *(E,E)-nona-2,4-dienal* contribute to a green and fatty flavor in sweet potatoes. Overall, the sweet potato aroma is predominantly attributed to aldehydes and terpenoids, which contribute to flower, sweet, tallow, green, and fat flavors.

### 2.3. Correlation Analysis of Aroma and Content of Starch, Soluble Sugar, and Carotenoid in Five Sweet Potato Cultivars

**Figure 3:**
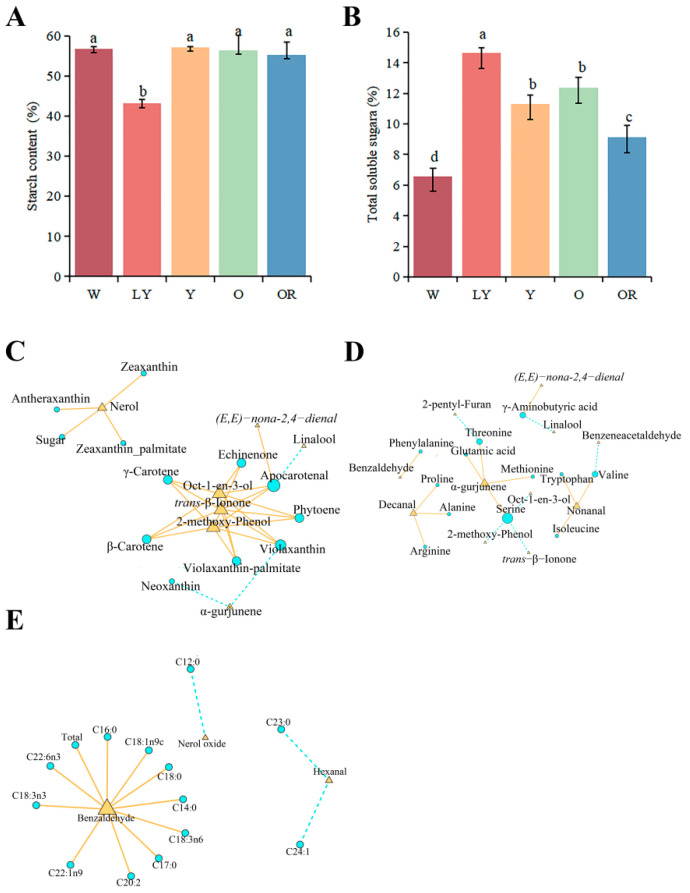
Starch (**A**) and soluble sugar (**B**) contents of five sweet potato cultivars. Lowercase letters a–d represent significance at *p* ≤ 0.05. Error bars represent mean ± SD (*n* = 3). (**C**) Correlation analysis of total starch content, soluble sugar content, and carotenoids content with characteristic aromas. (**D**) Correlation analysis between amino acids and characteristic aromas. (**E**) Correlation analysis between fatty acids and characteristic aromas. The blue circle represents the aroma precursors and the yellow triangle represents the characteristic aromas. (r ≥ 0.80 or −0.80, *p* < 0.05).

Carbohydrates are one of the main precursors of VOCs. Therefore, we determined and analyzed the starch and soluble sugar of five sweet potato cultivars. The results showed the dry matter content of the five sweet potato cultivars were as follows: 30.92% (W), 28.89% (LY), 36.92% (Y), 28.46% (O), and 31.89% (OR). Additionally, the starch content based on dry weight was determined, with Y exhibiting the highest value at 57.20%, followed by W at 56.77%, O at 56.47%, OR at 55.31%. Notably, LY had significantly lower starch content of only 43.15% (Figure 3A). The soluble sugar content based on dry weight for these five sweet potato cultivars ranged from 14.65–6.59%. Among them, LY exhibited the highest value while W had the lowest value; however, there were no significant differences observed between O and Y which recorded values of 12.38% and 11.29%, respectively. Furthermore, the followed OR showed a soluble sugar content of 9.11% (Figure 3B). In addition, carotenoids from five sweet potato cultivars have been detected [3]. To gain a comprehensive understanding of the relationship between different substances and aroma, we performed correlation network analysis on the levels of total starch, soluble sugar, carotenoids, and aroma (Figure 3C). The results revealed no significant correlation between starch content and key aromatic compounds. However, a statistically significant positive correlation was observed between soluble sugar and nerol (r ≥ 0.80 and *p* < 0.05). Carotenoids showed significant associations with various terpenoids and aldehydes. Among them, the contents of seven carotenoids exhibited a significant positive correlation with Oct-1-en-3-ol, 2-pentyl-Furan, and *trans*-β-ionone (r ≥ 0.80 and *p* < 0.05). The contents of antheraxanthin, zeaxanthin, and zeaxanthin palmitate exhibited a significant positive correlation with nerol (r ≥ 0.80 and *p* < 0.05). The concentration of apocarotenal exhibited a significant negative correlation with linalool (r ≥ 0.80 and *p* < 0.05), while it showed a significant positive correlation with *(E,E)-nona-2,4-dienal* (r ≥ −0.80 and *p* < 0.05). In addition, the contents of alpha-gurjunene exhibited a significant negative correlation with neoxanthin and violaxanthin (r ≥ −0.80, *p* < 0.05).

### 2.4. Correlation Analysis of Aroma with Amino Acids Contents in Five Sweet Potato Cultivars

As the precursor of volatile organic compounds, amino acids can affect the taste of food to a certain extent. In our work, twenty amino acids were detected and quantified by HPLC-MS/MS in the five sweet potato cultivars. The results showed there were significant differences in amino acid contents among the five sweet potatoes. The variety W exhibited the highest total amino acid content (1794.78 μg/g), followed by Y with 1605.45 μg/g, OR with 1440.46 μg/g, and LY with 1391.80 μg/g. Among them, O had the lowest amino acid content of 1058.51 μg/g (Table 2). Aspartic acid and glutamic acid were found to be the most abundant amino acids in all five sweet potatoes, with aspartic acid having the highest content in O at 426.98 μg/g, accounting for 40.34% of the total content. Followed by asparagine and glutamine, the most abundant in W, 443.71 μg/g and 139.88 μg/g, respectively, were twice as many as the other four sweet potatoes. It was worth noting the content of phenylalanine was the highest among the three aromatic amino acids (126.82–62.15 μg/g), followed by tyrosine (35.34–16.99 μg/g), and the content of tryptophan was the lowest (19.11–8.18 μg/g). In addition, six human essential amino acids were detected, threonine was the most abundant in Y (108.83 μg/g), and the content of the other five amino acids, valine, methionine, isoleucine, leucine, and lysine, was about 20 μg/g. Most of the remaining amino acids are lowest in O (Table 2). Through the correlation analysis between amino acid contents and key aromatic compounds, we identified a significant correlation between 12 amino acids and 11 aroma compounds (Figure 3D). Serine exhibited a negative correlation with Oct-1-en-3-ol, 2-pentyl-Furan, and *trans*-β-Ionone (r ≥ −0.80 and *p* < 0.05). Alpha-gurjunene showed a significant positive correlation with glutamic acid, methionine, threonine, and serine (r ≥ 0.80 and *p* < 0.05). Furthermore, our results revealed a significant positive correlation (r ≥ 0.80, *p* < 0.05) between phenylalanine and benzaldehyde.

### 2.5. Correlation Analysis of Aroma with Fatty Acids Contents in Five Sweet Potato Cultivars

Fatty acids are also important precursors of VOCs. To understand the relationship between fatty acids and VOCs of different sweet potatoes, we determined the fatty acids of five cultivars and analyzed their correlation with VOCs. A total of 25 fatty acids were detected and quantified. The results revealed significant variations in total fatty acid content among different cultivars, with Y exhibiting the highest total fatty acid content (6.45 μg/mg) among the five sweet potato cultivars. The other cultivars had slightly lower levels: OR (5.30 μg/mg), W (4.13 μg/mg), and LY (3.57 μg/mg). O had the lowest total fatty acid content at 3.55 μg/mg (Table 3). The main fatty acid components of sweet potatoes were C16:0 and C18:0, accounting for 42–48% and 25–29% of the total content, respectively. Followed by C18:2n6c, accounting for 13–15%. In contrast, the remaining fatty acids content is minuscule. The correlation analysis revealed a significant association between only three key aromatic compounds and thirteen fatty acids (Figure 3E). Among them, the levels of benzaldehyde exhibited a significant positive correlation with 10 fatty acids (r ≥ 0.80 and *p* < 0.05). Furthermore, a significantly negative correlation was observed between nerol oxide and C12:0 (r ≥ −0.80, *p* < 0.05). Additionally, hexanal showed a negative correlation with C23:0 and C24:1 (r ≥ −0.80, *p* < 0.05).

## 3. Discussion

VOCs are secondary metabolites of biological regulatory processes and their accumulation in plants is related to carbohydrate, amino acid, and fatty acid metabolism [24]. However, the main chemical components in sweet potato and their relationship to VOCs have not been reported [25]. Therefore, we identified the VOCs, starch, soluble sugar, amino acid, and fatty acid and measured their contents in five sweet potato cultivars and conducted correlation analysis. In our study, half of the VOCs in five sweet potato cultivars were terpenoids, followed by aldehydes, which was consistent with our previous findings [19,24]. In addition, to better understand the contribution of VOCs to sweet potato aroma, we calculated the ROAV value of VOCs. The results showed there were 15 compounds with ROAV > 1, mainly aldehydes and terpenoids, which provided the sweet potato with a flower, sweet, tallow, green, and fat, taste.

The synthesis of terpenoids has been predominantly attributed to two factors: (1) glycosylation of sugars catalyzed by glycosyltransferase, leading to the production of terpenoids [26]. In our results, soluble sugar content was significantly positively correlated with nerol, which provided a rich sweet for sweet potato. (2) The carotenoid degradation process primarily yields terpenoids, which are derivatives of carotenoids. Werner et al. found β-carotene could degrade to formβ-ionone [27]. In our study, we observed a significant positive correlation between carotenoid contents and Oct-1-en-3-ol, 2-pentyl-Furan, as well as *trans*-β-Ionone. Notably, *trans*-β-ionone was exclusively detected in O and OR samples while the highest β-carotene content was found in these two groups. Based on these findings, we postulated β-carotene served as the primary precursor for sweet potato *trans*-β-Ionone production which contributed to the unique floral characteristics of this crop.

The aldehydes, as a pivotal class of aromatic compounds, has been found to be intricately linked to the degradation processes of amino acids, fatty acids, and other substances, particularly phenylalanine, linolenic acid, and linoleic acid. In our study, a total of 20 amino acids and 25 fatty acids were identified, among which phenylalanine, linoleic acid, and linolenic acid exhibited the highest content in Y. The correlation analysis revealed significant associations between 12 amino acids and 13 fatty acids with key aromatic compounds of sweet potato. It is worth noting phenylalanine and 10 kinds of fatty acids were significantly positively correlated with benzaldehyde. This is due to the fact that in plants, phenylalanine, serving as the primary precursor for the biosynthesis of volatile styrene, undergoes a series of 11 enzymatic reactions to enter the β-oxidation pathway. And, forms the immediate precursor of benzene compounds, ultimately leading to the degradation into benzaldehyde [28]. At the same time, phenylalanine was converted into either phenylacetaldehyde or phenylpyruvic acid by the carbon group generated during lipid oxidation, which was subsequently degraded by free radicals produced during lipid hydroperoxide decomposition to form benzaldehyde [29]. In addition, the levels of C23:0 methyl tricosanoate and C24:1 methyl nervonate exhibited a significant positive correlation with hexana. Previous studies have demonstrated caproaldehyde was generated through β-oxidative degradation of long chains synthesized via the lipoxygenase (LOX) pathway [14].

Therefore, the key aromatic compounds terpenoids and aldehydes are present in all five sweet potato cultivars. The synthesis of terpenoids is closely linked to the breakdown of soluble sugars and carotenoids, which contribute to the sweet and flower taste of sweet potatoes. Similarly, the synthesis of aldehydes is associated with the degradation of amino acids and fatty acids, imparting a fat and green flavor profile to sweet potatoes.

## 4. Materials and Methods

### 4.1. Plant Material

Five raw sweet potatoes Shangshu 19 with white flesh (W), Okinawa-No. 100 with light-yellow flesh (LY), Jieshu 95-16 with yellow flesh (Y), Guangshu 87 with orange flesh (O), and Pushu 32 with orange-red flesh (OR) were planted with standard agricultural practices in Baiyun Experimental Station (23°23′ N, 113°26′ E; 20 m above sea level), Guangdong Academy of Agricultural Sciences in Guangzhou, China.

About 100 g was retrieved from 3–5 medium-sized sweet potatoes from each variety. They were rinsed with distilled water and allowed to air dry. The raw sweet potato samples were divided into two parts, one of which was ground into fine powder, a crop-fresh sample, with a liquid nitrogen grinder and stored at −80 °C. The other portion of the sample was subjected to freeze-drying for 72 h, followed by grinding into a fine powder. Subsequently, it was securely stored in a sealed bag at −20 °C.

### 4.2. Determination of Volatile Organic Compounds by HS-SPME/GC-MS

The volatile organic compounds (VOCs) present in fresh sweet potato samples were analyzed using the HS-SPME/GC-MS technique. Precisely measured 1 g of fresh sweet potato sample was transferred into a 15 mL glass bottle. Then, 2 mL of saturated NaCl solution and 0.5 L of 1-heptanol (0.137 μg mL^−1^, CAS: 111-70-6) were added. The sweet potato samples were pre-treated at 100 °C for 30 min to ensure full cooking, followed by incubation at 70 °C for 30 min and subsequent extraction at the same temperature for another 30 min. The GC-MS analysis method and data processing were based on the methodology described by Yao et al. [30]. VOCs were identified by linear retention index (RI) of n-alkanes assay and query mass spectrometry of the Data System Library (NIST 2017). The relative content of VOCs was calculated using the formula VOCs = (peak area of VOCs/peak area of 1-heptanol) × 0.137 μg mL^−1^.

### 4.3. Relative Odor Activity Value Analysis

The relative odor activity of the compounds was calculated based on the threshold value and relative concentration of the compounds in water, and the contribution of the compounds to sweet potato aroma was estimated by ROAV. ROAV = relative concentration/odor threshold of the compound. When ROAV is >1, it indicates the compound has a direct contribution to the aroma and is identified as a key aromatic compound [20].

### 4.4. Determination of Total Starch

The determination of total starch contents was conducted utilizing the Total Starch Assay Kit. (Megazyme, Wicklow, Ireland) [31].

### 4.5. Determination of Soluble Sugar Content

The soluble sugar content was determined using the anthrone colorimetric method, following the protocol described by Maness et al. [32]: weighing 100 mg of freeze-dried powder in a tube and performing two extractions using 80% ethanol. Finally, 3 mL of anthranone for color development was added and the absorbance at 620 nm was measured.

### 4.6. Determination of Amino Acids by HPLC-MS/MS

The samples, weighing fifty milligrams each, were subjected to extraction using 0.6 mL of 0.1 M hydrochloric acid for a duration of one hour with gentle agitation on a shaker at room temperature [33]. The sample was passed through a 0.22 μm pore membrane filter, followed by the addition of 10 μL of the filtered sample into a UHPLC vial containing 70 μL Borate buffer and 20 μL AccQ·Tag reagent. The reaction mixture was incubated at room temperature for 1 min, subsequently heated to 55 °C for a duration of 10 min, and finally subjected to injection of 1 μL after cooling.

The sample extracts underwent analysis utilizing a UPLC-Orbitrap-MS system (Thermo Fisher Scientific, Waltham, MA, USA). The analytical conditions were as followed for UPLC analysis: the column used was Waters ACQUITY UPLC BEH C18 (1.7 μm, 50 × 2.1 mm) with a column temperature of 55 °C. The flow rate was set at 0.5 mL/min and the injection volume was 1 μL. The solvent system consisted of water (0.1% Formic acid) and acetonitrile (0.1% Formic acid). A gradient program was employed with the following ratios: 95:5 *v*/*v* at 0 min, 90:10 *v*/*v* at 5.5 min, 75:25 *v*/*v* at 7.5 min, 40:60 *v*/*v* at 8 min, and finally returning to the initial ratio of 95:5 *v*/*v* at both minute points 8.5 and 13. The HRMS data were acquired using a Q Exactive hybrid Q-Orbitrap mass spectrometer equipped with a heated ESI source (Thermo Fisher Scientific, Waltham, MA, USA) and employing the Full MS acquisition method. The ESI source parameters were configured as followed: spray voltage was set to 3 kV; sheath gas pressure was adjusted to 40 arb; aux gas pressure was regulated at 10 arb; sweep gas pressure remained at 0 arb; capillary temperature was maintained at 320 °C; and aux gas heater temperature was controlled at 350 °C. The amino acid identification was conducted through a comprehensive analysis of retention times, co-chromatography with pure standards, and quantification using external calibration (Table S1).

The data were acquired on the Q-Exactive instrument using Xcalibur 4.1 software (Thermo Fisher Scientific, Waltham, MA, USA) and subsequently processed with TraceFinder™4.1 Clinical software (Thermo Fisher Scientific, Waltham, MA, USA). The quantified results were exported in excel format.

### 4.7. Determination of Fatty Acids by GC-MS

Reconstituted 100 mg samples in a sterile EP tube, was followed by the addition of 4 mL of chloroform. Subsequently, we employed vortexing for a duration of 30 s to ensure thorough mixing [34]. We reconstituted the sample by adding a 0.9% NaCl solution and vortexing for 30 s, centrifuged at 3500 rpm for 15 min at ambient temperature, followed by transferring the lower layer of liquid to a separate test tube. Subsequently, we introduced 2 mL of dichloromethane, vortexed for 30 s, centrifuged for another 15 min, and subsequently discarded the bottom layer. We combined the lower liquid and dried it using nitrogen gas. Subsequently, we introduced 2 mL of methanol (containing 5% sulfuric acid), vortexed for a duration of 30 s, and subjected the sample to a water bath at 80 °C for a period of 2 h. After cooling in the water bath, we added 2 mL of n-hexane and 1 mL of water. We vortexed the mixture for 30 s, followed by centrifugation at a speed of 2000 rpm for 5 min. After collecting the supernatant, we added 1 mL of water and vortexed for 30 s. we centrifuged at 2000 rpm for 5 min, followed by collection of the supernatant which was subsequently dried using nitrogen gas. We added 0.5 mL of isooctane, adjusted based on the sample concentration, and vortexed for 30 s. The sample was allowed to stand for 5 min before transferring the solution to the sample bottle for detection.

The gas chromatography system used in this study was the Agilent 7820 model manufactured by Agilent Technologies, Santa Clara, CA, USA. According to the compound properties, a CP-Sil 88 gas chromatographic column (100 m × 0.25 mm × 0.25 μm, Agilent, Santa Clara, CA, USA) was employed for analysis. The injection volume was set at 1 L with a split ratio of 10:1, while high purity helium was employed as the carrier gas at a flow rate of 1.0 mL/min. The column oven was initially set at a temperature of 100 °C for a duration of 5.0 min, followed by a programmed ramp to reach 240 °C at a rate of 4 °C/min over the course of 15 min. The carrier gas, helium, was maintained at a constant flow rate of 1 mL min−1 through the column. The injector temperature was set to 260 °C. The mass spectral analysis was conducted using selected ion monitoring, employing ions determined by a standard for each compound, with a quadrupole temperature of 150 °C and a fragmentation voltage of 70 eV. The identification of fatty acids was based on a comprehensive analysis of retention times, co-chromatography with pure standards, and quantification through external calibration (Table S2).

Data were acquired on the MassHunter GC/MS Acquisition (Agilent Technologies, Santa Clara, CA, USA) and processed using Quant-My-Way (Agilent Technologies, Santa Clara, CA, USA).

### 4.8. Data Processing and Statistics

The samples were subjected to triplicate analysis and the results were expressed as mean ± standard deviation (SD). One-way ANOVA and T-test were performed using SPSS v.26.0 (IBM, SPSS Inc., Chicago, IL, USA) to determine significant differences. The criterion for statistical significance was set at a significance level of *p* < 0.05. The TBtools software (TBtools GUI v0.66443) was utilized for the construction of unsupervised principal component analysis (PCA) charts and heatmaps, while Log2-based expression folding changes were performed. The correlation network diagram was constructed using Cytoscape software (Cytoscape v3.7.2) (r ≥ 0.80 or −0.80 and *p* < 0.05).

## 5. Conclusions

In this study, the VOCs of five sweet potato cultivars were analyzed, OAV values were calculated, and the characteristic aroma substances of sweet potato were identified. Additionally, the aroma precursor substances including total starch content, soluble sugar content, amino acid content, and fatty acid content were determined and examined. The results revealed the detection of a total of 66 VOCs in the five sweet potatoes, with terpenoids accounting for 59% and aldehydes comprising 17% of the identified VOCs. Among these, the most abundant VOCs were found in OR. Based on relative odor activity value (ROAV) calculations, we selected fifteen key aromatic compounds including seven aldehydes, six terpenes, and one each of furan and phenol to contribute to the flower, sweet, tallow, green, and fat taste profiles of sweet potatoes. We identified a total of 20 amino acids in sweet potato, with glutamic acid, aspartate, glutamine, asparagine, and phenylalanine being the most predominant ones. In terms of fatty acids, we detected 25 different types, among which linolenic acid and linoleic acid exhibited high abundance. The results of aroma and precursor correlation analysis revealed a positive correlation between soluble sugar and nerolol, while seven carotenoids exhibited a positive correlation with *trans*-β-Ionone, which provided sweet potato with flower fragrance. Furthermore, phenylalanine and 10 types of fatty acids demonstrated significant correlations with benzaldehyde, providing a burnt sugar flavor to sweet potatoes. And, two fatty acids exhibit a significant association with hexana, thereby imparting its distinct fat flavor profile. In summary, by screening the characteristic aroma compounds of sweet potato and conducting correlation analysis, it was discovered the secondary metabolism of sweet potato aroma precursors contributed to its rich flavor profile and provided a valuable foundation for future processing and application of sweet potato.

## Figures and Tables

**Table 1:** The odor activity value (ROAV ≥ 1) of volatile compounds detected in five sweet potato cultivars.

Aroma Compounds	Class	Threshold (mg/kg) ^a^	Description ^b^	W	LY	Y	O	OR
Oct-1-en-3-ol	aldehydes	0.002	mushroom				9.11	14.18
Hexanal	aldehydes	0.0045	grass, tallow, fat	19.15	15.28	7.09	8.89	22.77
Benzaldehyde	aldehydes	0.05	almond, burnt sugar	1.22	1.08	2.06	1.25	1.52
Benzeneacetaldehyde	aldehydes	0.04	hawthorne, honey, sweet	1.72	1.34	1.40	1.99	1.82
Nonanal	aldehydes	0.0035	fat, citrus, green	6.46	10.02	5.25	4.27	3.80
Decanal	aldehydes	0.003	soap, orange peel, tallow	10.37	10.00	7.08		7.40
*(E,E)-nona-2,4-dienal*	aldehydes	0.00006	fat, wax, green					41.10
2-pentyl-Furan	furan	0.0048	Green bean, butter	3.88	7.05	3.51	6.71	9.59
Guaiacol	phenols	0.00017	smoke, sweet, medicine				95.90	114.44
Linalool	terpenoids	0.0015	flower, lavender	40.37	21.92	32.15	7.31	
Nerol oxide	terpenoids	0.007	oil, flower		35.85		4.46	
Nerol	terpenoids	0.049	sweet		9.96	3.57	2.06	0.28
Geraniol	terpenoids	0.0075	rose, geranium	8.64	49.92	10.99	15.01	2.50
*trans*-β-Ionone	terpenoids	0.000461	seaweed, violet, flower, raspberry				70.73	175.63
alpha-gurjunene	terpenoids	0.001	wood, balsamic	47.95	19.04	72.06	5.62	11.23

^a^ Odor characteristics and threshold values of related compounds in water, referring to the relevant literature Jiang et al. [20,21,22,23]. ^b^ Description: https://www.flavornet.org/flavornet.html (accessed on 2 October 2023).

**Table 2:** Amino acid content (µg/g on fresh weight basis) in five sweet potato cultivars.

	W	LY	Y	O	OR
Aspartic acid	398.07 ± 0.06 ab	378.58 ± 0.05 cd	422.54 ± 0.03 ab	426.98 ± 0.01 a	388.80 ± 0.02 bc
Glutamic acid	368.55 ± 0.06 b	246.36 ± 0.05 d	407.38 ± 0.03 a	193.08 ± 0.01 e	286.10 ± 0.03 c
Asparagine	443.71 ± 0.02 a	211.26 ± 0.07 c	176.04 ± 0.04 d	78.61 ± 0.02 e	244.05 ± 0.03 b
Glutamine	139.88 ± 0.02 a	100.32 ± 0.06 b	61.54 ± 0.05 c	50.01 ± 0.01 d	105.8 ± 0.04 b
Phenylalanine	62.15 ± 0.02 d	72.16 ± 0.06 bc	126.82 ± 0.02 a	67.95 ± 0.01 c	75.93 ± 0.01 b
Tyrosine	16.99 ± 0.02 d	35.34 ± 0.06 a	27.30 ± 0.03 b	18.44 ± 0.02 d	24.25 ± 0.01 c
Tryptophan	11.94 ± 0.01 b	19.11 ± 0.06 a	10.90 ± 0.04 bc	8.18 ± 0.01 d	9.73 ± 0.03 c
Threonine	58.53 ± 0.03 b	37.86 ± 0.06 c	108.83 ± 0.03 a	33.47 ± 0.01 cd	32.04 ± 0.01 d
Valine	24.81 ± 0.04 bc	32.46 ± 0.08 a	25.64 ± 0.03 b	19.42 ± 0.02 d	22.56 ± 0.01 c
Methionine	20.51 ± 0.01 a	16.53 ± 0.07 b	20.15 ± 0.03 a	11.49 ± 0.02 d	15.09 ± 0.01 c
Isoleucine	16.21 ± 0.03 b	24.95 ± 0.08 a	15.38 ± 0.02 b	12.65 ± 0.02 c	14.21 ± 0.01 bc
Leucine	18.37 ± 0.03 b	21.98 ± 0.07 a	16.10 ± 0.04 c	11.48 ± 0.02 d	15.48 ± 0.01 c
Lysine	6.38 ± 0.07 b	5.96 ± 0.03 b	9.06 ± 0.04 a	4.95 ± 0.03 c	8.93 ± 0.01 a
Histidine	19.73 ± 0.49 a	15.08 ± 0.027 b	12.27 ± 0.44 c	8.69 ± 0.08 d	12.94 ± 0.34 c
Serine	93.75 ± 0.03 a	79.69 ± 0.06 b	81.01 ± 0.03 b	57.60 ± 0.01 c	62.48 ± 0.01 c
Glycine	14.81 ± 0.02 a	13.85 ± 0.09 a	9.7 ± 0.03 b	8.96 ± 0.07 b	9.67 ± 0.01 b
Arginine	25.75 ± 0.02 a	20.46 ± 0.07 b	18.05 ± 0.05 c	10.74 ± 0.06 d	27.71 ± 0.01 a
Alanine	33.99 ± 0.05 b	30.29 ± 0.08 c	34.25 ± 0.03 b	19.57 ± 0.02 d	40.36 ± 0.01 a
Gamma-Aminobutyric acid	5.34 ± 0.07 d	8.88 ± 0.06 b	6.92 ± 0.02 c	5.43 ± 0.04 d	24.21 ± 0.05 a
Proline	14.32 ± 0.03 b	19.74 ± 0.07 a	14.69 ± 0.03 b	9.01 ± 0.02 c	18.76 ± 0.01 a
Total	1793.78	1390.86	1604.58	1056.73	1439.10

Different letters represent significant (*p* < 0.05) differences between means according to ANOVA combined with Duncan’s multiple range test. Each value represents the mean ± standard deviation (*n* = 3).

**Table 3:** Content (µg/mg on fresh weight basis) in five sweet potato cultivars.

	W	LY	Y	O	OR
C10:0	8.88 × 10^−3^ ± 0.015 b	0.88 × 10^−3^ ± 0.03 e	16.87 × 10^−3^ ± 0.02 a	1.44 × 10^−3^ ± 0.02 d	2.81 × 10^−3^ ± 0.03 c
C12:0	7.89 × 10^−2^ ± 0.01 b	1.33 × 10^−2^ ± 0.03 d	9.77 × 10^−2^ ± 0.02 a	1.56 × 10^−2^ ± 0.02 d	4.21 ± 0.03 c
C14:0	2.53 × 10^−2^ ± 0.02 c	1.94 × 10^−2^ ± 0.03 e	4.54 × 10^−2^ ± 0.02 a	0.02 × 10^−2^ ± 0.02 d	0.03 ± 0.04 b
C15:0	9.84 × 10^−3^ ± 0.06 c	5.33 × 10^−3^ ± 0.03 e	12.83 × 10^−3^ ± 0.06 a	0.01 ± 0.02 d	0.01 ± 0.01 b
C14:1	2.51 × 10^−3^ ± 0.02 b	3.22 × 10^−3^ ± 0.05 b	6.22 × 10^−3^ ± 0.12 a	2.91 × 10^−3^ ± 0.06 b	3.37 × 10^−3^ ± 0.06 b
C16:0	1.83 ± 0.01 c	1.56 ± 0.02 e	2.75 ± 0.02 a	1.70 ± 0.01 d	2.27 ± 0.03 b
C17:0	6.80 × 10^−3^ ± 0.01 c	6.85 × 10^−3^ ± 0.04 c	11.66 × 10^−3^ ± 0.03 a	6.66 × 10^−3^ ± 0.04 c	8.08 × 10^−3^ ± 0.06 b
C18:0	1.12 ± 0.013 c	0.93 ± 0.03 e	1.66 ± 0.02 a	1.03 ± 0.01 d	1.42 ± 0.03 b
C18:1n9c	0.02 ± 0.03 d	0.03 ± 0.03 bc	0.08 ± 0.05 a	0.03 ± 0.04 cd	0.04 ± 0.16 b
C18:2n6c	0.61 ± 0.01 c	0.55 ± 0.03 d	0.87 ± 0.03 a	0.48 ± 0.02 e	0.79 ± 0.04 b
C18:3n3	0.02 ± 0.01 c	0.02 ± 0.03 c	0.04 ± 0.05 a	0.02 ± 0.02 c	0.03 ± 0.05 b
C18:3n6	1.86 × 10^−3^ ± 0.02 c	1.61 × 10^−3^ ± 0.07 cd	4.04 × 10^−3^ ± 0.04 a	1.43 × 10^−3^ ± 0.10 d	2.21 × 10^−3^ ± 0.05 b
C20:0	2.78 × 10^−3^ ± 0.04 c	5.28 × 10^−3^ ± 0.08 b	8.98 × 10^−3^ ± 0.03 a	2.25 × 10−3 ± 0.07 c	5.15 × 10^−3^ ± 0.04 b
C20:1	0.09 ± 0.019 d	0.10 ± 0.03 c	0.15 ± 0.03 b	0.07 ± 0.02 e	0.17 ± 0.04 a
C20:2	2.63 × 10^−3^ ± 0.04 c	3.07 × 10^−3^ ± 0.02 bc	5.27 × 10^−3^ ± 0.11 a	2.47 × 10^−3^ ± 0.05 c	3.71 × 10^−3^ ± 0.06 b
C20:3n6	0.75 × 10^−3^ ± 0.04 d	1.22 × 10^−3^ ± 0.03 c	2.93 × 10^−3^ ± 0.06 a	0.94 × 10^−3^ ± 0.03 d	1.53 × 10^−3^ ± 0.02 b
C20:5n3	8.96 × 10^−3^ ± 0.01 c	9.29 × 10^−3^ ± 0.03 c	17.83 × 10^−3^ ± 0.07 a	6.86 × 10^−3^ ± 0.04 d	12.68 × 10^−3^ ± 0.02 b
C21:0	0.01 ± 0.02 c	0.01 ± 0.02 c	0.03 ± 0.06 a	0.01 ± 0.03 d	0.02 ± 0.06 b
C22:0	0.26 ± 0.02 c	0.28 ± 0.02 c	0.60 ± 0.02 a	0.12 ± 0.02 d	0.40 ± 0.04 b
C22:1n9	4.12 × 10^−3^ ± 0.01 c	4.18 × 10^−3^ ± 0.02 c	9.39 × 10^−3^ ± 0.06 a	3.86 × 10^−3^ ± 0.06 c	0.01 ± 0.05 b
C22:2	3.15 × 10^−3^ ± 0.03 d	4.15 × 10^−3^ ± 0.01 c	8.67 × 10^−3^ ± 0.03 a	2.05 × 10^−3^ ± 0.03 e	5.72 × 10^−3^ ± 0.03 b
C22:6n3	0.01 ± 0.03 c	0.01 ± 0.02 c	0.01 ± 0.07 a	0.01 ± 0.04 c	0.01 ± 0.05 b
C23:0	0.23 × 10^−3^ ± 0.08 c	0.43 × 10^−3^ ± 0.01 bc	1.41 × 10^−3^ ± 0.15 a	5.14 × 10^−3^ ± 0.07 b	0.31 × 10^−3^ ± 0.06 bc
C24:0	2.90 × 10^−3^ ± 0.01 d	3.61 × 10^−3^ ± 0.01 c	6.74 × 10^−3^ ± 0.03 a	1.75 × 10^−3^ ± 0.07 e	4.67 × 10^−3^ ± 0.04 b
C24:1	0.26 × 10^−3^ ± 0.08 c	0.26 × 10^−3^ ± 0.12 c	1.51 × 10^−3^ ± 0.05 a	0.43 × 10^−3^ ± 0.18 b	0.16 × 10^−3^ ± 0.36 c
Total	4.13	3.57	6.45	3.55	5.30

Different letters represent significant (*p* < 0.05) differences between means according to ANOVA combined with Duncan’s multiple range test. Each value represents the mean ± standard deviation (*n* = 3).

## Data Availability

We will provide data upon request.

## Supplementary Materials

The following supporting information can be downloaded at: https://www.mdpi.com/article/10.3390/molecules29030606/s1, Table S1: Calibration curves of amino acid standard. Table S2: Calibration curves of fatty acid standard. Table S3: Composition and content of volatile organic compounds in five sweet potato cultivars.
